# Bioinformatics analyses of potentially common pathogenic networks for primary Sjögren’s syndrome complicated with acute myocardial infarction

**DOI:** 10.1038/s41598-023-45896-5

**Published:** 2023-11-06

**Authors:** Qingbin Hou, Jinping Jiang, Kun Na, Xiaolin Zhang, Dan Liu, Quanmin Jing, Chenghui Yan, Yaling Han

**Affiliations:** 1 Department of Internal Medicine (Cardiovascular), the Second Clinical Medical College, Shanxi Medical University, Taiyuan, China; 2 State Key Laboratory of Frigid Zone Cardiovascular Disease, Cardiovascular Research Institute and Department of Cardiology, General Hospital of Northern Theater Command, Shenyang, China; 3 Department of Cardiology, Shengjing Hospital Affiliated to China Medical University, Shenyang, China

**Keywords:** Immunology, Biomarkers

## Abstract

Both primary Sjögren’s syndrome (pSS) and acute myocardial infarction (AMI) are intricately linked. However, their common mechanism is not fully understood. Herein, we examined the underlying network of molecular action associated with developing this complication. Datasets were downloaded from the GEO database. We performed enrichment and protein–protein interaction analyses and screened key genes. We used external datasets to confirm the diagnostic performance for these hub genes. Transcription factor and microRNA regulatory networks were constructed for the validated hub genes. Finally, drug prediction and molecular docking validation were performed. We identified 62 common DEGs, many of which were enriched regarding inflammation and immune response. 5 DEGs were found as key hub genes (IGSF6, MMP9, S100A8, MNDA, and NCF2). They had high diagnostic performance in external datasets. Functional enrichment of these five hub genes showed that they were associated with the adaptive immune response. The Type 1T helper cell showed the most association among all cell types related to AMI and pSS. We identified 36 common TFs and 49 identical TF-miRNAs. The drugs, including Benzo, dexamethasone, and NADP, were predicted as potential therapeutic agents. Herein, we revealed common networks involving pSS and AMI etiologies. Knowledge of these networks and hub genes can enhance research into their associated mechanism and the development of future robust therapy.

## Introduction

Primary Sjögren's syndrome (pSS) is a classic chronic and systemic autoimmune disease, with clinical manifestations including oral and eye dryness, fatigue, and joint pain^1^. The clinical symptomology can be complex, diverse, and heterogeneous. pSS can easily damage critical organs and negatively impact human health. The present global pSS incidence rate is 0.3–1/1000^2,3^. Several prior investigations revealed that inflammatory/autoimmune diseases (namely, systemic lupus erythematosus and psoriasis) increase the prevalence of cardiovascular events among pSS patients^4–6^. Being a relatively recent autoimmune disease, pSS is similar to the aforementioned autoimmune diseases, particularly in pathogenesis and clinical manifestations^7^. It was reported that pSS patients experience an enhanced risk of cardiovascular events^8^. Emerging reports suggest that pSS patients without prior cardiovascular disease (CVD) display subclinical atherosclerotic tendencies, namely, endothelial dysfunction and early onset arterial wall damage^9^. Bartoloni et al.^10^ compared 800 adult female pSS patients with 4500 healthy controls and revealed that hypertension and hypercholesterolemia incidences were markedly enhanced among pSS patients compared to controls. Wu Xue Fen et al.^11^ reported that pSS is a stand-alone risk factor for coronary heart disease following the exclusion of traditional Framingham risk factors, namely hypertension, diabetes, and hyperlipidemia.

Given the above reports, there are available data on the clinical correlation of pSS and CVD with metabolic syndrome, atherosclerosis, and other chronic diseases. However, acute myocardial infarction (AMI) is a primary form of CVD that endangers human health, and its morbidity and mortality rates are on the rise. Till now, there have been limited investigations on pSS and AMI.

**Figure 1:**
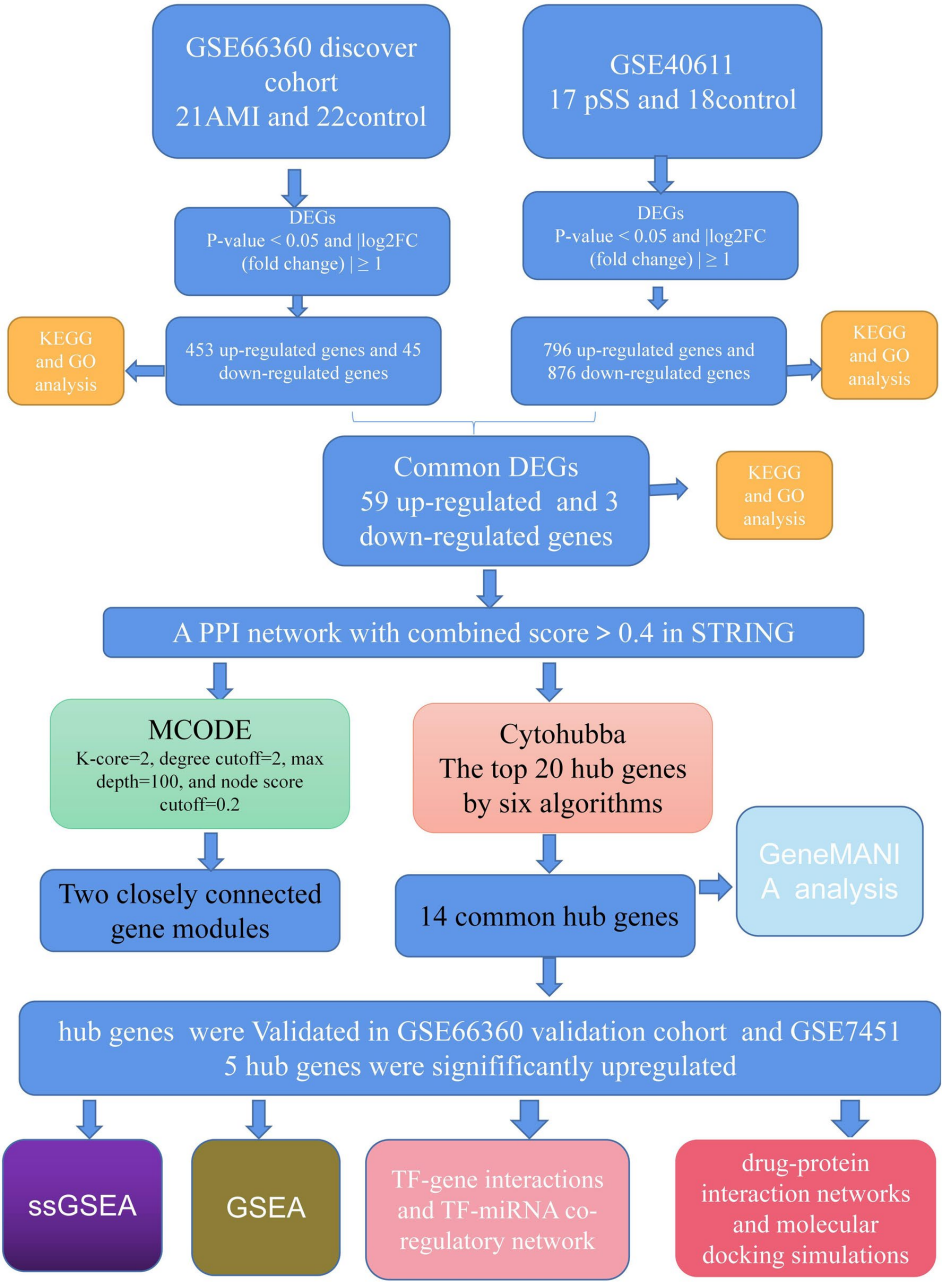
Research design flow chart.

Herein, we screened the available pSS and AMI datasets in the GEO database (as discover cohort, DC) for common differentially expressed genes (C-DEGs) and assessed their physiological activities. Using the STRING database, we generated the protein–protein interaction (PPI) network, and using Cytoscape, we screened for hub genes. Our results were further validated using a validated cohort (VC). Using Gene Set Enrichment Analysis(GSEA), we performed functional analyses of the selected hub genes. Subsequently, we identified these DEGs' TF-gene interaction and TF-miRNA co-modulatory networks. Potential therapeutic agents were screened through drug-protein interaction networks and molecular docking simulations. As mentioned above, our conclusions provide new ideas for further elucidation and genetic associations of the two diseases. The study design is illustrated in Fig. 1.

## Results

### Screening of AMI- and pSS-based DEGs

**Figure 2:**
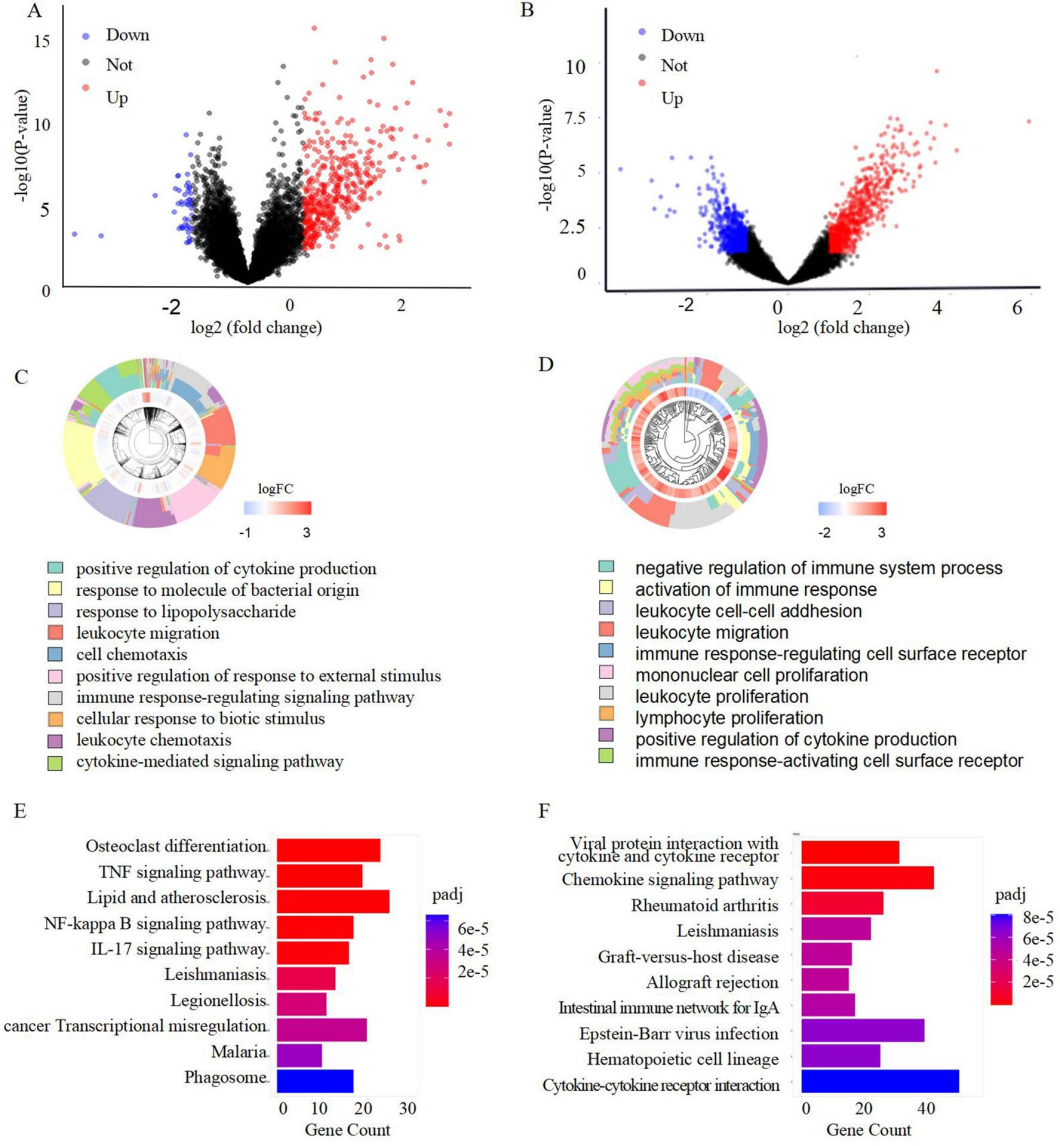
Screening DEGs for AMI and pSS, respectively. (**A**) Volcano plot revealing 498DEGs between AMI and control group. (**B**) Volcano plot indicating 1672 DEGs between pSS and control group. (**C**) The GO-BP terms in AMI. (**D**) The GO-BP terms in pSS. (**E**) The KEGG analysis of AMI DEGs.(F)The KEGG analysis of pSS DEGs.

In the GSE66360 DC, we observed 453 highly- and 45 scarcely-expressed genes between the AMI patients and controls (Fig. 2A). Moreover, there were 796 highly- genes and 876 scarcely-expressed genes between the pSS patients and healthy controls in the GSE40611 dataset (Fig. 2B). Gene Ontology (GO) analysis: the DEG enrichments were in response to lipopolysaccharides, direct modulation of cytokine synthesis, leukocyte migration, cell chemotaxis, and immune response-modulating axis (Fig. 2C). In the pSS dataset, GO enrichment was seen in the immune response, mononuclear and leukocyte proliferation and migration, and immune response-modulating cell surface receptor (Fig. 2D). Our KEGG (Kyoto Encyclopedia of Genes and Genomes) (https://www.kegg.jp/kegg/kegg1.html) analysis revealed that the AMI dataset enriched the inflammatory networks, lipids and arteriosclerosis, immune response, and parasitic diseases (Fig. 2E). Alternately, the pSS dataset showed enrichment in the immune, inflammatory, and viral invasive networks (Fig. 2F).

### Screening of C-DEGs

**Figure 3:**
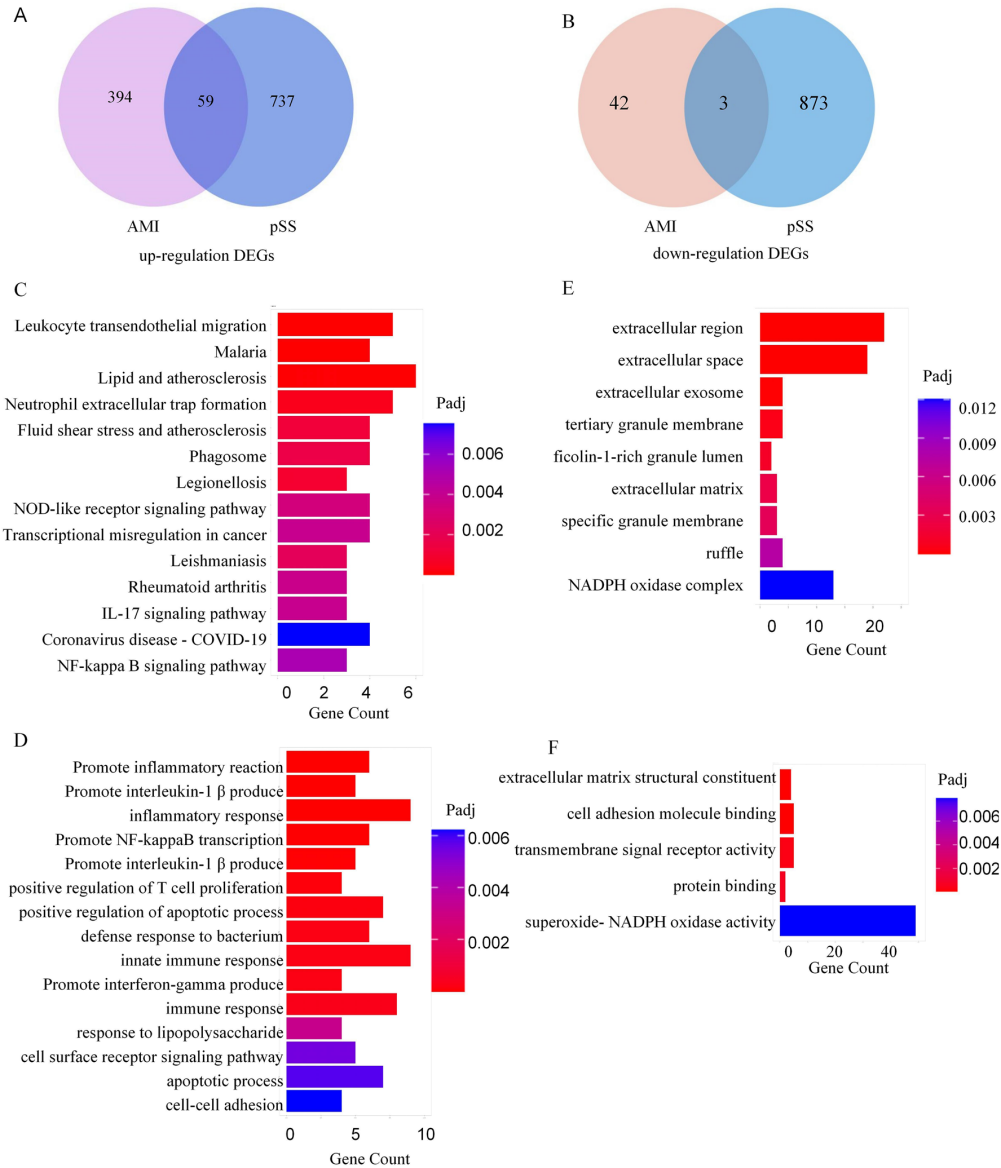
Identification and Functional Enrichment Analysis of C-DEGs. (**A**) Venn diagram revealing 59 Up-regulated common DEGs in AMI and pSS. (**B**) Venn diagram demonstrating 3 Down-regulated common DEGs in AMI and pSS. (**C**) KEGG pathway analysis of C-DEGs. (**D**) GO-BP analysis of C-DEGs. (**E**) GO-CC analysis of C-DEGs. (**F**) GO-MF analysis of C-DEGs.

Our Venn Diagram of DEGs between the GSE66360 DC and GSE40611 dataset revealed 62 C-DEGs. Among them, 59 were markedly enhanced (Fig. 3A), and 3 were drastically reduced (Fig. 3B). Information on the C-DEGs is summarized in Supplementary Table 1.

### Evaluation of the functional profiles of C-DEGs

In terms of KEGG enrichment analysis (https://www.kegg.jp/kegg/kegg1.html) (Fig. 3C), the C-DEGs showed enrichment in the leukocyte transendothelial migration (Padj = 3.6 × 10^–5^), lipid and atherosclerosis (Padj = 0.7 × 10^–4^), neutrophil extracellular trap formation (Padj = 0.4 × 10^–3^), legionellosis (Padj = 2.2 × 10^–3^), fluid shear stress and atherosclerosis (Padj = 1.2 × 10^–3^). The KEGG analysis results are summarized in detail in Supplementary Table 2.

In terms of the GO biological process (BP) enrichment analysis (Fig. 3D), C-DEGs showed enrichment in the direct modulation of an inflammatory response (Padj = 1.3 × 10^–6^), direct modulation of interleukin-1 beta synthesis (Padj = 1.9 × 10^–6^), inflammatory response (Padj = 5.5 × 10^–6^), fungus detection (Padj = 9.8 × 10^–6^), direct modulation of NF-kappaB transcription factor activity (Padj = 1.2 × 10^–5^), direct modulation of interleukin-6 synthesis (Padj = 1.9 × 10^–5^), acute inflammatory response (Padj = 3.3 × 10^–5^), MyD88-dependent toll-like receptor axis (Padj = 4.4 × 10^–5^), direct modulation of T cell proliferation (Padj = 5.3 × 10^–5^), and direct modulation of apoptosis (Padj = 8.1 × 10^–5^). In the case of the GO -CC and GO-MF enrichment analyses (Fig. 3E, F), the C-DEGs showed enrichment in the extracellular region (Padj = 7.80 × 10^–8^), extracellular space (Padj = 1.80 × 10^–6^), and extracellular matrix (ECM) structural constituent (Padj = 7.60 × 10^–6^). The GO results are summarized in detail in Supplementary Table 3. These findings suggest that activation of the traditional inflammatory pathway causes leukocyte migration by developing extracellular traps by neutrophils, proliferation of T cells, and acceleration of the immune response through endothelial cells. Furthermore, enormous amounts of chemotactic cytokines and inflammatory mediators were produced, hastening the progression of both diseases.

### The protein–protein interaction (PPI) Axis Generation and Modular Analysis

**Figure 4:**
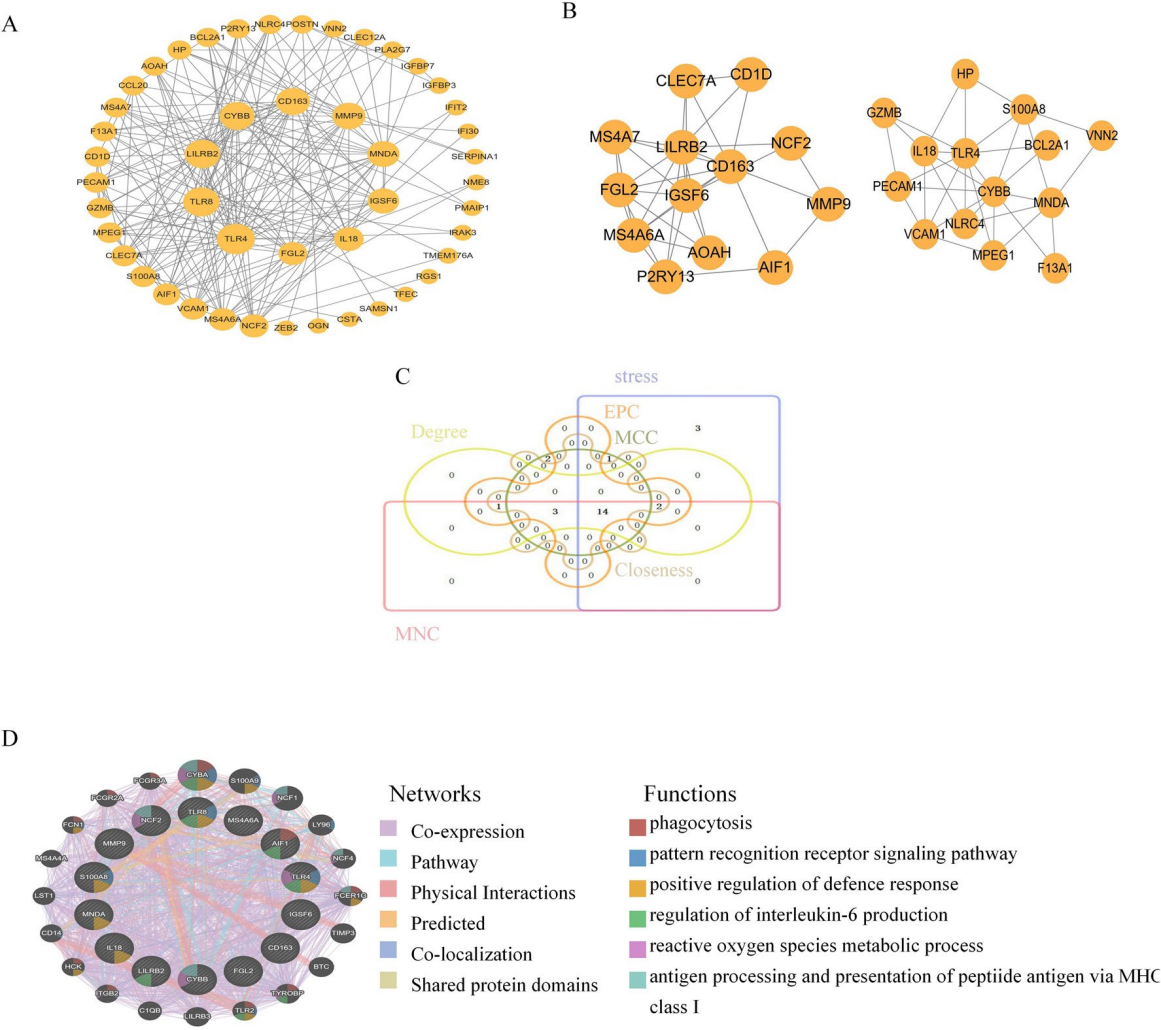
PPI network and Venn diagram, the co-expression network of hub genes. (**A**) PPI network of C-DEGs. (**B**) Significant gene module and enrichment analysis of the modular genes. (**C**) Common hub genes by six algorithms. (**D**) Hub genes and their co-expression genes were analyzed via GeneMANIA.

C-DEG data were entered into STRING for PPI axis generation. The subsequent results with a combined score > 0.4 were imported into Cytoscape to generate a PPI axis containing 38 nodes and 268 interaction pairs (Fig. 4A). We obtained 2 closely connected gene modules, which included 27 C-DEGs and 69 interaction pairs, using the MCODE plug-in of Cytoscape (Fig. 4B).

### Identification and assessment of hub genes

The 20 leading hub genes were identified using 6 algorithms of the cytoHubba plug-in (Supplementary Tables 4, 5). By intersection, 14 C-DEGs, namely, TLR8, LILRB2, TLR4, IGSF6, CYBB, CD163, MNDA, FGL2, MS4A6A, IL18, MMP9, AIF1, NCF2, and S100A8 were identified (Fig. 4C). Subsequently, employing the GeneMANIA database, we assessed the co-expression networks and associated roles of hub genes. The PPI axis revealed a co-expression of 74.98%, physical interactions of 9.73%, networks of 10.04%, co-localization of 2.37%, estimated 2.82%, and shared protein domains of 0.06% (Fig. 4D).

### Verification of hub gene expression

**Figure 5:**
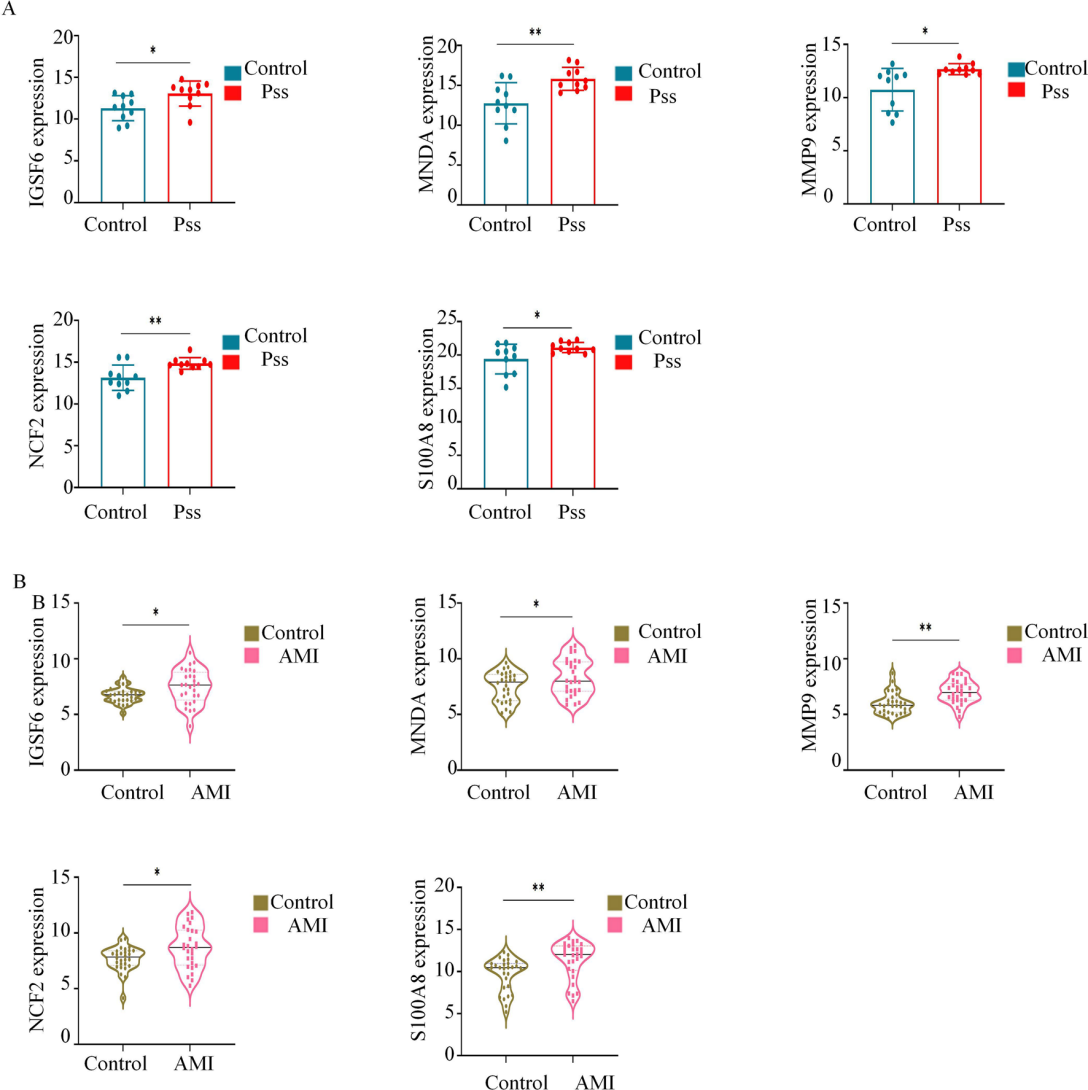
Validation of hub genes. (**A**) IGSF6, MNDA, MMP9, NCF2 and S100A8 were validated in GSE7405. (**B**) IGSF6, MNDA, MMP9, NCF2 and S100A8were validated in GSE66360 validation cohort . *p < 0.05, **p < 0.01.

To identify genes essential for AMI and pSS cooccurrence, we validated the hub genes for C-DEGs in the GSE66360 VC for AMI (Fig. 5A) and GSE7451 (Fig. 5B) for pSS from GEO. Based on our results, only 5 hub genes were strongly enhanced in both AMI and pSS samples, relative to controls. These hub genes were IGSF6, S100A8, MNDA, MMP9, and NCF2.

### GSEA of hub genes, relationship between hub genes and immune invasion

**Figure 6:**
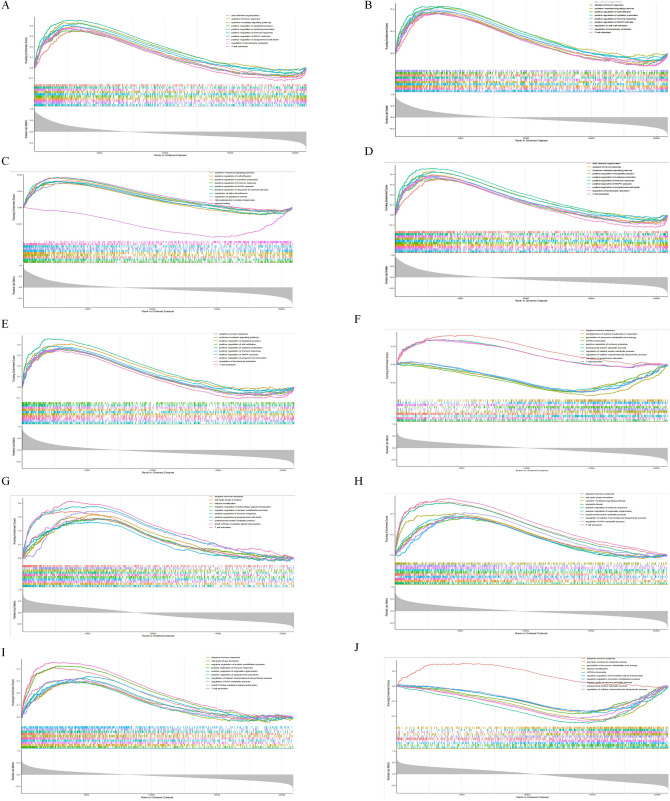

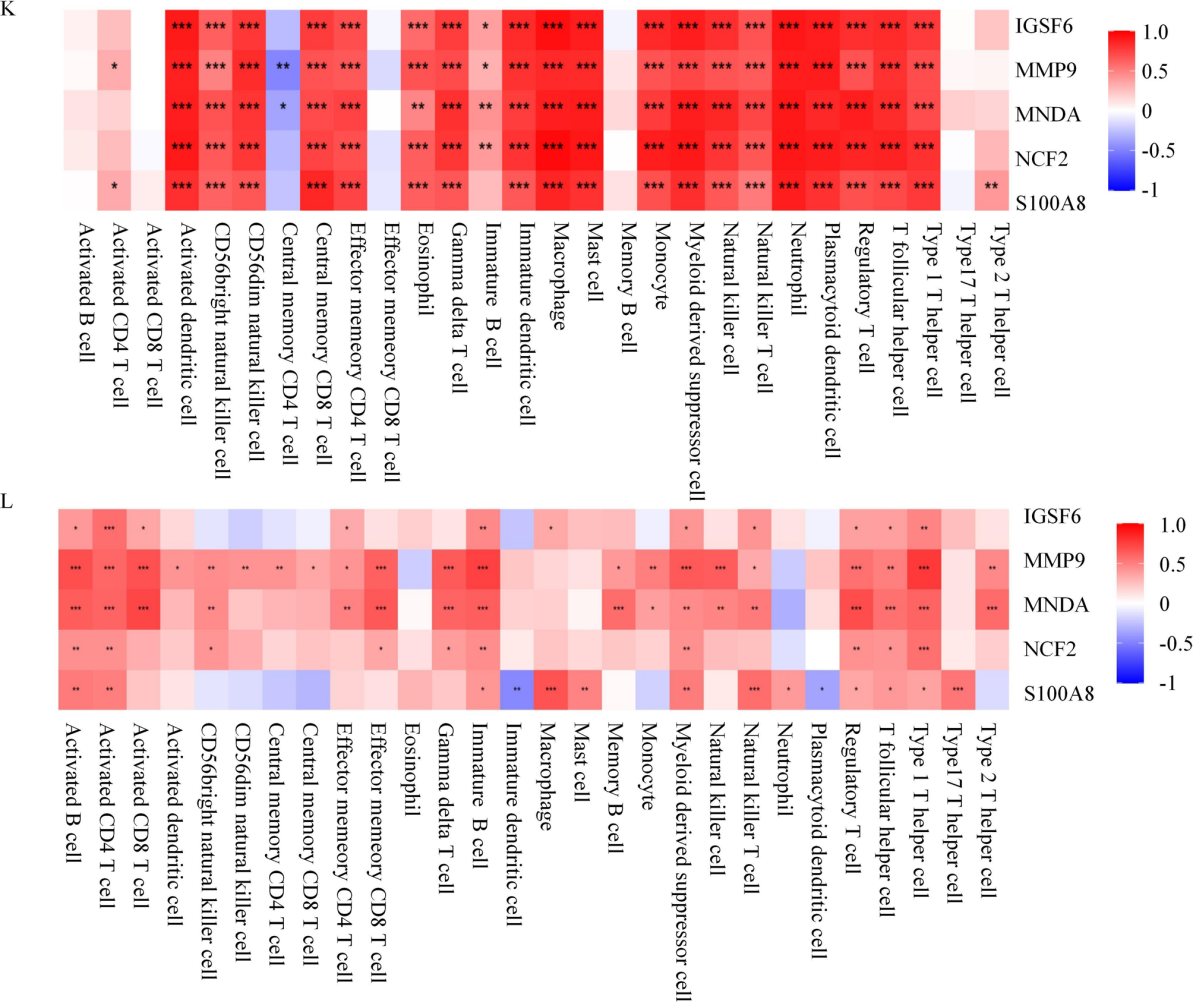
Gene set enrichment analysis (**A**–**E**), the association between the hub genes and immune infiltration: A merged enrichment plot of IGSF6, MNDA, MMP9, NCF2, and S100A8 in GSE66360 discover cohort. (**F**–**J**) An integrated enrichment plot of IGSF6, MNDA, MMP9, NCF2, and S100A8 from gene set enrichment analysis in GSE40611. (**K**) In the GSE66360 discover cohort, S100A8, IGSF6, MNDA, NCF2, and MMP9 positively correlate with most cell types. (**L**) In theGSE40611 dataset, S100A8, IGSF6, MNDA, NCF2 and MMP9 were shown to correlate positively with many cell types. Red: positive correlation; blue: negative correlation.

To elucidate the potential physiological roles of the 5 identified hub genes between pSS and AMI, we performed GSEA. We revealed that the adaptive immune response was strongly associated with enhanced IGSF6, MMP9, S100A8, MNDA, and NCF2 expressions in GSE66360 DC (Fig. 6A–E) and GSE40611 (Fig. 6F–J).

We used ssGSEA to evaluate possible relationships between the 5 hub genes found and the 28 immune cells. In GSE66360 DC, these 5 hub genes were directly associated with most cellular types,as shown in Fig. 6K. In the GSE40611 dataset (Fig. 6L), we observed that the S100A8, IGSF6, MNDA, NCF2, and MMP9 were directly associated with activated CD4 T cell, activated B cell, immature B cell, myeloid-derived suppressor cell, type 1T helper cell, T follicular helper cell, regulatory T cell, type 1T helper cell, and T follicular helper cell. Upon combining the ssGSEA results of the datasets mentioned above, we identified the myeloid-derived suppressor cell, Type 1T helper cell, T follicular helper cell, regulatory T cell, and Type 1T helper cell were associated with AMI and pSS. Among them, the Type 1T helper cell was the most relevant cell type.

### TF-gene associations and TF-miRNA co-modulatory axis

**Figure 7:**
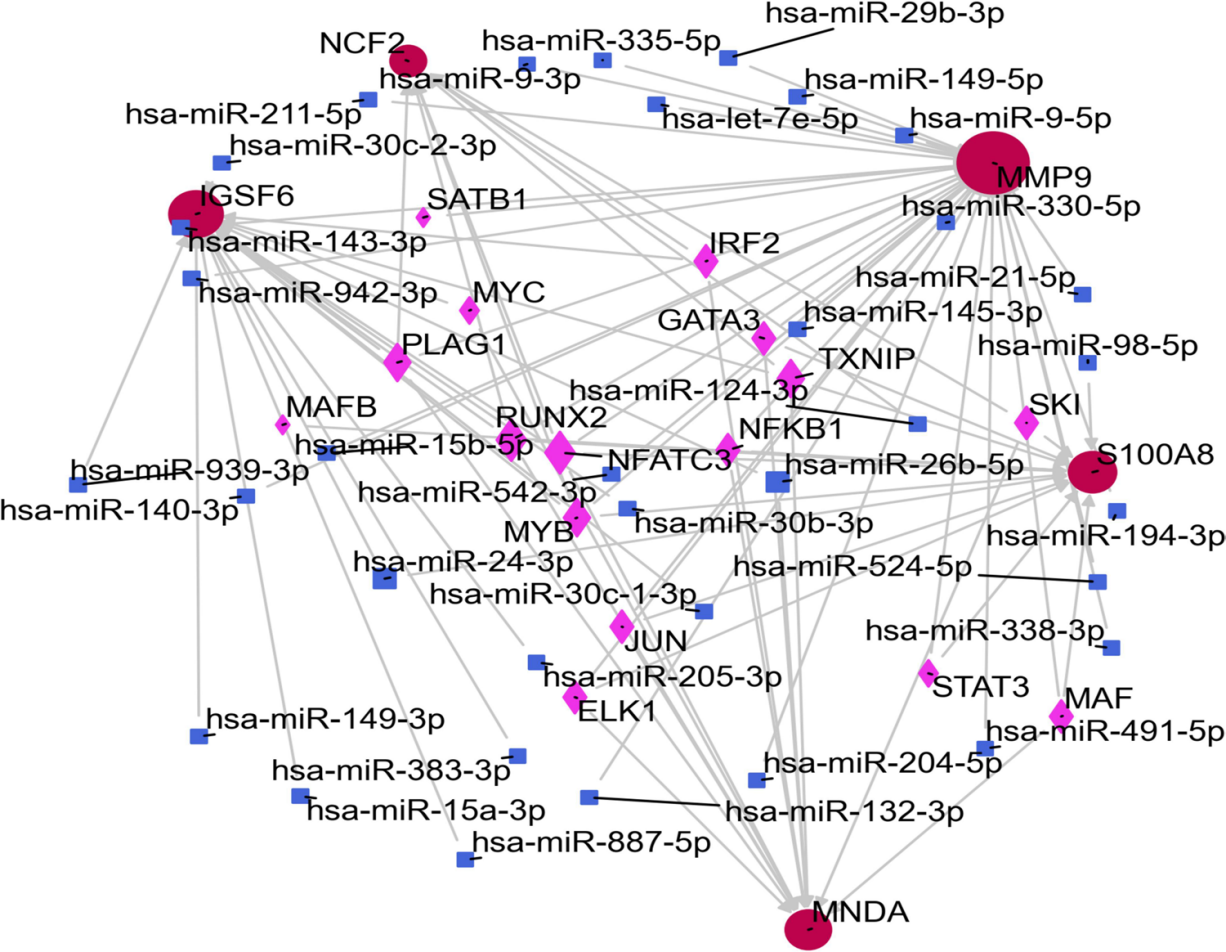
The network presents the TF-miRNA coregulatory network. Red: common genes, pink: TF genes, blue: miRNA.

Next, we used Networkanalyst to construct the TF miRNA co-modulatory axis using NetworkAnalyst to estimate the association between miRNAs + TFs and hub genes. We combined the experimentally validated database TF-Knock v2.0 with the miRTarBase screening subnetwork to validate our predicted TF-miRNA network. We screened potential TFs from the TF-Knock database that were knocked down to affect ‘MNDA’, ‘MMP9’, ‘IGSF6’, ‘S100A8’, ‘NCF2’. In the miRTarBase database, we screened potential miRNAs whose binding has been experimentally confirmed by western blot, clip-seq, micro-array, and other methods. Our network revealed 55 nodes and 85 edges, including 36 miRNA-based interactions with hub genes and 49 TF-hub genes (Fig. 7). These associations are likely to modulate the overall hub gene expression.

### Identification of candidate drugs and targeted chemical interactions in AMI and pSS

Chemical-protein interaction networks are essential research tools for understanding protein functions and advancing drug discovery. Using the C- DEGs in AMI and pSS, candidate drugs were identified using Enrichr based on the DSigDB database. The top 10 drug molecules were selected according to their p-values and were considered potential drug candidates. These 10 candidate drugs included Benzo, 9,12-octadecadienoic acid, dexamethasone, phorbol 12-myristate 13-acetate, acetoacetic acid, NADP, chromium(VI), Acrolein, sulfaguanidine, and arachidonic acid (Supplementary Table 6). These candidate drugs reacted with the common DEGs, indicating they were applicable for treating both diseases.

**Figure 8:**
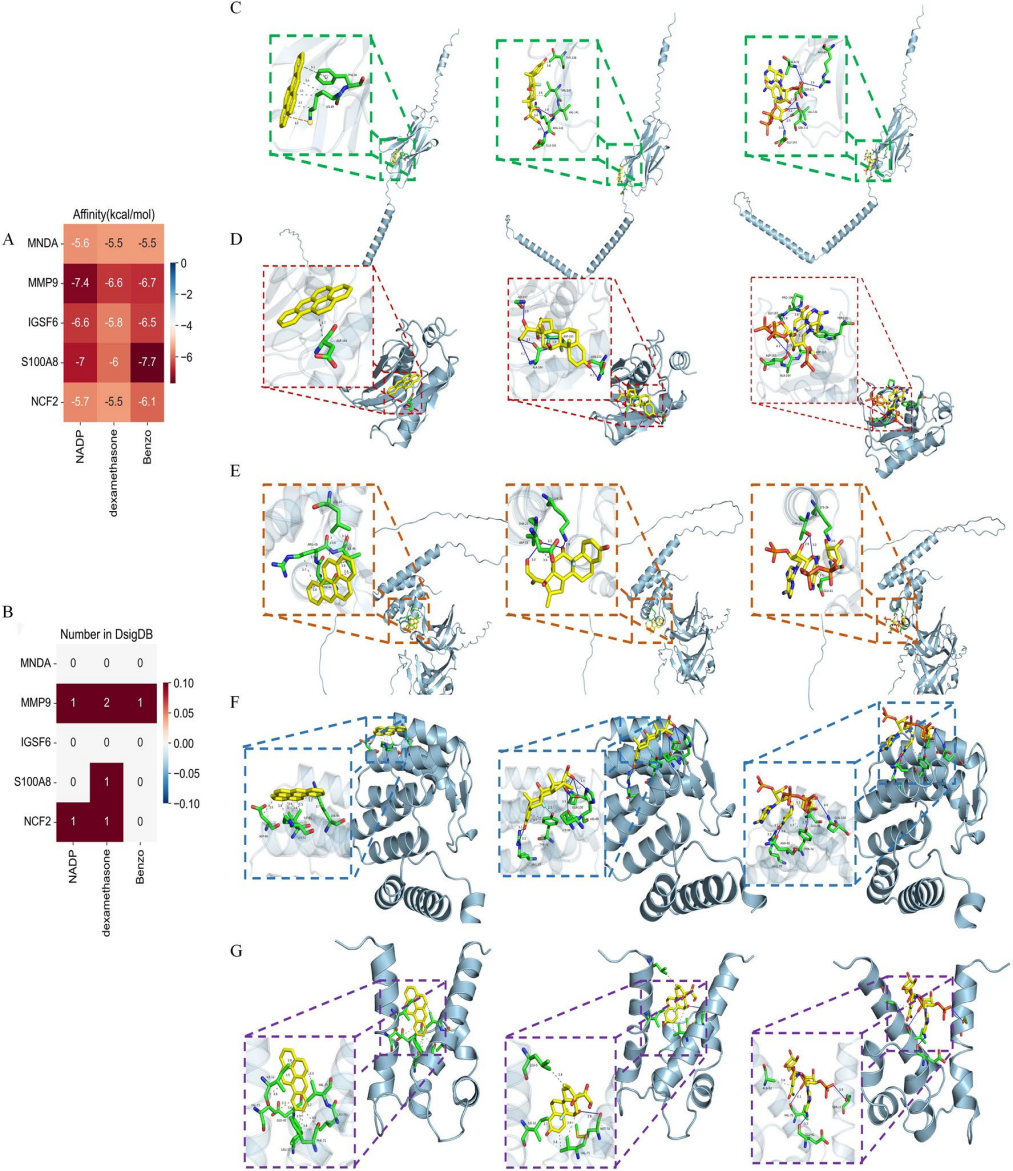
Molecular docking patterns. (**A**) Heatmap showing the affinity between molecular and target gene. (**B**) Heatmap showing the number of interactions between known molecules and target genes in the DSigDB database. (**C**) Molecular docking pattern for IGSF6 (**C**), MMP9 (**D**), MNDA (**E**), NCF2 (**F**), and S100A8 (**G**). The molecules in order from left to right are Benzo, dexamethasone, and NADP.

Furthermore, molecular docking was used to predict the binding modes of drugs with hub genes, namely, IGSF6, MMP9, S100A8, MNDA, and NCF2 (Supplementary Table 7). We calculated the binding affinity of each of the three molecules to five target genes (Fig. 8A). In addition to this, we validated our identified molecule-target gene pairs using the DSigDB database, which is an experimentally validated molecule-target gene database (Fig. 8B). Not all molecule-target genes are found in experimentally validated databases, and our predictions identified Benzo, den, and NADP as not only capable of binding MMP9 and NCF2, with similar binding affinities suggesting the ability to bind MNDA IGSF6 as well. The molecular docking results are shown in Fig. 8C–G. Interestingly, Benzo, dexamethasone, and NADP were found to have lower stabilization energies at their binding sites to the five target proteins. Therefore, these three candidate drugs may have the potential for thebe the treatment of both AMI and pSS.

With enormous advancements in recent medical technology, the autoimmune disease detection rate and the standard of care have greatly improved^12^. In addition, numerous investigations explore correlations between various frequently occurring diseases^13^. CVD remains a significant disease endangering public health worldwide^14,15^. One study reported that patients with autoimmune diseases experienced a 56% elevated CVD rate (23.3 per 1000 person-years) compared to the general population (15.0 per 1000 person-years). All CVD patients demonstrated an enhanced CVD risk with autoimmune disease, and the risk was progressive with the number of autoimmune diseases present (one disease: HR 1.41 [95% CI 1.37–1.45]; two diseases: 2.63 [2.49–2.78]; three or more diseases: 3.79 [3.36–4.27]). Moreover, this reported increase in CVD risk is difficult to explain using currently known cardiovascular risk factors^16^. However, further mechanistic research is still restricted to autoimmune diseases like rheumatoid arthritis and systemic lupus erythematosus, which are known to increase CVD incidence^4–6^ vastly. In the case of pSS, there were reports of comparable effects on well-known autoimmune disease pathology. However, its relation to CVD is relatively unknown. One report revealed that pSS patients are 0.08 times more likely to experience a cardiovascular event than healthy controls^16^. Moreover, pSS patients reported significantly enhanced risks of cerebrovascular events and MI^10^. Nevertheless, the associated mechanism is undetermined. Herein, we identified the C-DEGs between pSS and MI and explored the physiological roles and networks related to the C-DEGs.

We demonstrated 59 highly- and 3 scarcely-expressed genes among the C-DEGs between pSS and AMI datasets. Using GO analysis, The AMI dataset was strongly enriched in the migration and chemotaxis of myeloid and granulocyte cells following lipopolysaccharide and bacterial stimulation. In the pSS dataset, the GO enrichment analysis showed enrichment in the immune response and mononuclear and leukocyte proliferation and migration. Based on our KEGG analysis, the AMI dataset was enriched in the inflammatory networks, lipids and arteriosclerosis, immune response, and parasitic diseases.In contrast, the pSS dataset enhanced the immune, inflammatory, and viral invasion networks. We next employed the C-DEGs to form a PPI axis to search for hub genes. We identified 5 hub genes, namely, IGSF6, MNDA, MMP9, NCF2, and S100A8. Our GSEA analysis revealed hub gene enrichment in the adaptive immune response, whereas ssGSEA showed the most considerable correlation with the Type 1T helper cell.

S100A8 is a member of the EF-hand calcium-interacting protein S100 family and modulates multiple cellular processes. During a pathological-induced inflammatory response, neutrophils and monocytes are the initial innate immune cells to reach the inflammation site and release S100A8^17–19^. S100A8/A9 modulates adaptive immune response and is reported to induce phagocyte hyporesponsiveness to LPS stimulation via a methyltransferase G9a-based chromatin modification in a NF-κB and TLR-4 dependent fashion^20^. S100A8 is known to modulate pSS^21^and MI etiologies, as well as MI/reperfusion (I/R) injury^22^.

MMP9 belongs to the MMPs family and is ubiquitously expressed within neutrophils and macrophages. Upon stimulation, the Type 1T helper cell surface CD40L is up-regulated, which, in turn, enhances MMP9 synthesis. MMP9 hydrolyzes the ECM and augments inflammatory cell migration, particularly those of macrophages and neutrophils to the ECM. The MMP-9-induced elimination of 4 CXCL6N-terminal residues up-regulates CXCL6 biological activity. The modified CXCL12 enhances macrophage differentiation to pro-angiogenic and immunosuppressive cell types, suggesting that MMP-9 regulates the direct coordination of macrophage polarity^23–25^.

MMP9 enhances new blood vessel formation by increasing smooth muscle migration and VEGF release, which eventually weakens the fiber cap by degrading ECM while simultaneously improving inflammatory response by increasing monocytes/macrophages swelling, which strongly diminishes plaque stability, thereby encouraging thrombosis formation^26–28^. Clinical trials revealed that the MMP-9 content in the salivary and lacrimal glands of pSS patients is strongly enhanced^29^.

Neutrophil cytoplasmic factor 2 (NCF2) is a nicotinic adenine dinucleotide phosphate oxidase (NAPDHO) complex component. It synthesizes massive quantities of superoxide, which is transported to phagosomes for destruction^30^. Furthermore, it is reported that the NCF2 gene mutation is strongly associated with autoimmune diseases and is the pathogenic gene governing chronic granulomatous diseases, systemic lupus erythematosus, and other autoimmune diseases^31^.NCF2 is ubiquitously expressed among atherosclerotic patients^32^.

MNDA (human myeloid nuclear differentiation antigen) is found in specific hematopoietic cell lineages and is highly expressed in macrophages and neutrophils. It is central to a newly discovered nucleus-mitochondrion network that accelerates apoptosis. In addition, MNDA is reported to be a critical gene for predicting patients with systemic lupus erythematosus complicated with atherosclerosis^33^.

IGSF6 is named after a molecular domain much like an immunoglobulin and modulates cell adhesion. IGSF6 expresses on lymphocyte surface and controls immune response by modulating adhesion between lymphocytes and macrophages. The atherosclerotic etiology is associated with IGSF6^34^. However, there is no strong evidence regarding IGSF6 and pSS.

Herein, we used GSEA to reveal that the 5 identified hub genes strongly correlated with adaptive immune response. Using ssGSEA, we demonstrated that the hub genes were enriched in Type 1T helper cells. Hence, our conclusions corroborated with some of the reports mentioned above. We hypothesized that the Type 1T helper cell stimulates adaptive immune response to bring about pSS and AMI.

Type 1T helper cells modulate the immune response to protect against bacteria and protozoa. Among the associated cytokines, the most prominent is gamma interferon (IFN-γ) Th1, which corresponds to type 4 autoimmune disease^35–37^. IFN-γ has been extensively examined and was shown to modulate coordination between anti-inflammatory and immune responses. During pSS onset, IFN-γis up-regulated, which further worsens pSS development and progression^38^. IFN-γ is known to enhance Fas and CD40 contents in the SS salivary gland epithelial cells, which, in turn, accelerates salivary gland epithelial cell apoptosis. Studies revealed that IFN-γ destroys the submandibular gland via STAT1 activation. IFN-γ, synthesized within the glandular epithelium following inflammatory stimulation, increases Class HLA-II molecule expression, facilitating the presentation of autoantigens to lymphocytes. Activated lymphocytes secrete massive quantities of cytokines to increase the glandular epithelial cell-based expression of HLA-II molecules^38–40^. This, in turn, aggravates and perpetuates the autoimmune response, which leads to enhanced inflammation and epithelial damage. However, in acute MI patients, vascular plaque-based T lymphocytes release IFN-γ to alter vascular smooth muscle cell migration and proliferation. IFN-γstrongly inhibits the smooth muscle-based production of interstitial collagen and the activation of monocytes. This, in turn, degrades the ECM in the weak area of the plaque fiber cap, thereby destabilizing the plaque and inducing rupture. IFN-γpositively modulates myocardial cell necrosis by increasing local nitric oxide synthesis^41–44^.

At present, little is known about treating patients with acute myocardial infarction complicated with primary Sjogren’s syndrome, which is still the direction of unremitting efforts of experts and scholars in the industry. The cooperation between cardiologists and rheumatologists from different professional perspectives can better diagnose, treat, and prevent such complications. Our results suggested that Benzo, dexamethasone, and NADP could bind multiple critical targets associated with AMI and pSS, indicating that they may represent novel therapeutic drugs for these diseases. Dexamethasone is recognized as an essential treatment for Sjogren's syndrome. Several studies have found that dexamethasone can also be used for cardiovascular disease. Short-term use of dexamethasone rapidly reduces inflammation and relieves gout symptoms, with few adverse reactions. Dexamethasone also protects myocardial cells following myocardial infarction and may prevent or treat restenosis after endovascular stenting^45,46^. NADP is considered an essential co-factor in antioxidant defense and reductive biosynthesis, while Benzo has anti-inflammatory and analgesic effects. However, little is known about their role in the occurrence and development of AMI and pSS, and even less is known about their clinical use in such comorbidities.he pharmacological mechanisms underlying these therapeutic effects on pSS and AMI require further exploration.However, whether these drugs can be used in these diseases still needs more in-depth pharmacological experiments and solid clinical validation.

Our review of the published literature revealed limited investigations, particularly bioinformatic analyses, on the shared mechanism between AMI and pSS. Herein, we screened for C-DEGs, hub genes, and TFs of AMI and pSS to elucidate both AMI and pSS pathogeneses. However, our investigation had certain limitations. First, our work needs further verification using external validation; second, the hub gene functions require further exploration and in vitro model validation, which is the focus of our upcoming investigation.

In summary, we identified C-DEGs for AMI and pSS and performed enrichment and PPI network analyses to elucidate pathogeneses. We demonstrated that AMI and pSS share a pathogenic mechanism potentially mediated by specific hub genes. These specific hub genes may play a pathological role through Type 1T helper cells mediated adaptive immune responses. Our findings provide a possible direction for exploring the underlying mechanisms associated with AMI and pSS.

## Materials and methods

### Data collection

Using search terms “primary Sjogren’s syndrome” and “myocardial infarction,” we screened the entire GEO database^47^. The following datasets were included for analysis: First, those containing data from both case and control cohorts. Second, sequencing was done on the human species. Third, all datasets originated from identical sequencing platforms. Fourth, the total sample population of each dataset was over 20 cases to minimize data volatility. Fifth, the original dataset and the results were repeatable. We selected the GSE66360DC, GSE66360 VC, GSE40611, and GSE7451 data sets after intense screening. Among them, the GSE66360 DC contained 21 AMI and 22 control volunteer samples; the GSE66360 VC contained 28 AMI patients and 28 healthy volunteer samples; the GSE40611 dataset contained 17 pSS and 18 healthy volunteer samples; and the GSE7451 dataset consisted of 10 pSS and 10 healthy volunteer samples (Supplementary Table 8). We conducted log2 transformation on the genetic profile of DEGs and matched the probes with their gene symbols based on the annotation documentation of corresponding platforms. Lastly, we retrieved a gene matrix with rows representing sample names and columns meaning gene symbols for subsequent analyses.

### Screening of DEGs

The GEOquery and Limma packages in the R software were employed for DEG identification between the AMI and HC cohort in the GSE66360 DC and between HCs and pSS in the GSE40611 dataset. During probe ID conversion to gene ID, the probe ID was discarded if no corresponding gene ID was established. Upon conversion of multiple probe IDs to one gene ID, the median expression was considered the expression amount in the final computation. Significance was considered when the adjusted *p*-value was < 0.05 and |log2FC (fold change) |≥ 1.

### GO and KEGG analyses of DEGs

We conducted GO and KEGG (analyses https://www.kegg.jp/kegg/kegg1.html) to elucidate the physiological roles and functional associations of common pSS and AMI DEGs^48–51^. Significance was considered when the adjusted *p*-value was < 0.05. The clusterProfiler package in R was employed to visualize the leading 10 DEGs.

### Identification and enrichment analyses of C-DEGs

The Venn diagram package illustrated c-DEGs (both highly- and scarcely-expressed genes) from the GSE66360 DC and GSE40611. The GO and KEGG network analyses result from visualization was performed using the clusterProfiler package in R. Significance was considered at adjusted *p*-value < 0.05.

### PPI axis generation and modular analysis

Using the interactive gene search tool (http://string-db.org) (Version 11.0)^52^, we analyzed DEGs harboring the same patterns as identified in the previous analysis. Then, we obtained the up- or downstream modulation of direct combination or coexistence between the DEGs. Interaction with a comprehensive score > 0.4 was set as the significance threshold. We next imported the results from STRING to Cytoscape (http://www.cytoscape.org)^53^to generate a PPI axis. MCODE assesses key functional modules. criteria as follows: K-core = 2, degree threshold = 2, max depth = 100, and node score threshold = 0.2.

### Identification and analysis of hub genes

DEGs were screened using Cytoscape Hubba(version 3.4.0). In particular, we employed 6 algorithms (MNC, MCC, DMNC, degree, radiality, and stress) to identify hub genes. The 20 leading hub genes were visualized using a Venn diagram on an online website (http://jvenn.toulouse.inra.fr/app/example.html)^54^. A co-expression network of hub genes was generated using GeneMANIA^55^.

### Verification of hub gene expression in other datasets

We confirmed the transcript levels of the hub as mentioned above genes in the GSE66360 VC and GSE7451. The GSE66360 VC contained 28 AMI and 28 control samples, and the GSE7451 included 10 pSS and 10 control samples. We employed a t-test for inter-group assessment. *p* < 0.05.

### GSEA and immune invasion analysis

The previously identified hub genes were assessed via GSEA in R. GSEA evaluates gene distribution patterns from a pre-defined gene set in a list sorted by phenotypic relevance to determine its relevance to a given phenotype.

The ssGSEA score was employed to quantify immune cell invasion in AMI or pSS samples and establish the immune invasion status in the GSE66360 DC and GSE40611 datasets.

### TF-gene and TF-miRNA modulatory networks

TF-gene and TF-miRNA modulatory base networks were generated via the Networkanalyst platform (https://www.networkanalyst.ca/)^56^. TF-gene modulatory networks were filtered and verified by TF-Knock2.0 database (https://bio.liclab.net/KnockTFv2/search.php). We selected the blood as the tissue of interaction. TF-miRNA modulatory networks were filtered and verified by miRTarBase (https://mirtarbase.cuhk.edu.cn/~miRTarBase/miRTarBase_2022/php/search.php?), which binding has been experimentally confirmed by western blot, clip-seq, micro-array, and other methods.

### Evaluation of applicant drugs and molecular docking simulation

The web portal of Enrichr and the Drug Signatures Database (DSigDB) were used to analyze^57^. The crystal structures of these critical proteins were downloaded from the Protein Data Bank (https://www.rcsb.org/). All the docking experiments utilized the Autodock tools (version 1.5.4). The results were shown with binding energy.The heat map of binding energy is shown by Python’s seaborn (pythonversion3.9, seaborn 0.12).The final image is presented through Pymol (PyMOL Molecular Visualization System 2020).

## Conclusions

This study identified IGSF6, MMP9, S100A8, MNDA, and NCF2 as markers for the co-pathogenesis of AMI and pSS. Functional enrichment of these five hub genes is an adaptive immune response. The Type 1T helper cell showed the most association among all cell types related to AMI and pSS. We identified 27 common TFs and 20 identical TF-miRNAs. The drugs, including Benzo, dexamethasone, and NADP, were predicted as potential therapeutic agents for AMI and pSS. These biomarkers and the relationship of AMI and pSS with angiogenesis may help to provide a deeper understanding of the comorbidity of AMI with pSS.

### Supplementary Information

Supplementary Tables.

## Data Availability

The GSE40611, GSE7451and GSE66360 datasets are available in the GEO database (https://www.ncbi.nlm.nih.gov/geo/). The R script data used to support the findings of this study are included in the supplementary information file.

## Abbreviations

pSS: Primary Sjögren’s syndrome
AMI: Acute myocardial infarction
GEO: Gene expression omnibus
CVD: Cardiovascular disease
C-DEGs: Common differentially expressed genes
KEGG: Kyoto encyclopedia of genes and genomes
GO: Gene ontology
DC: Discover cohort
VC: Validated cohort
GSEA: Gene set enrichment analysis
