# Supplementary material for: Bioinformatics analyses of potentially common pathogenic networks for primary Sjögren’s syndrome complicated with acute myocardial infarction
Source: Sci Rep. 2023 Nov 6;13:19276. doi: 10.1038/s41598-023-45896-5 (PMC10630427; doi:10.1038/s41598-023-45896-5)
Supplement: Supplementary file 1 — Supplementary Tables. [file 41598_2023_45896_MOESM1_ESM.pdf]

Supplementary Material

Bioinformatics analyses of potentially common pathogenic networks for primary Sjögren's syndrome complicated with acute myocardial infarction

Qingbin Hou[1,2], Jinping Jiang[2,3], Kun Na[2], Xiaolin Zhang[2], Dan Liu[2], Quanmin Jing[2], Chenghui Yan[2*], Yaling Han[1,2*]

[1]Department of Internal Medicine (Cardiovascular), the Second Clinical Medical College, Shanxi Medical University, Taiyuan, China
[2] State Key Laboratory of Frigid Zone Cardiovascular Disease, Cardiovascular Research Institute and Department of Cardiology, General Hospital of Northern Theater Command, Shenyang, China
[3] Department of Cardiology, Shengjing Hospital affiliated to China Medical University, Shenyang, China
*Correspondence: Yaling Han e-mail:hanyaling@163.net.Chenghui Yan e-mail:yanch1029@163.com

Supplementary Tables

Table S1 Basic information for common DEGs

Gene symbol	Expression	GSE66360discover Log2(FC)	GSE40611 Log2(FC)
MPEG1	up	1.913305043	2.734547409
CD163	up	2.210267328	2.34617356
POSTN	up	2.159842941	2.072085254
CYBB	up	1.123544215	2.000647166
LGALS2	up	1.423684843	2.814083788
CD1D	up	1.379821879	2.5444166
CCL20	up	2.640077737	2.536622545
VCAM1	up	1.141932266	2.269979381
MMP9	up	2.183355593	2.251394389
VNN2	up	1.302645705	2.896467616

F13A1	up	1.500669007	1.682226091
MS4A7	up	1.253815674	1.812734803
GZMB	up	2.604074252	1.696105767
IFIT2	up	1.000678547	2.010572517
TLR8	up	1.794263321	2.102671565
SERPINA1	up	2.423116146	1.782567693
SAMSN1	up	1.031227354	2.438076307
PHACTR1	up	1.534149769	1.796343483
ZEB2	up	1.310476685	1.354200872
RTN1	up	1.57342956	1.550958302
PECAM1	up	1.002262505	1.557198605
CSTA	up	3.193884558	1.790750812
CPVL	up	2.199634022	2.283177898
CLEC12A	up	1.508820228	1.604390472
CH25H	up	1.772068679	1.17272492
BASP1	up	1.099572041	1.909085907
TLR4	up	2.103319836	1.745556641
PLA2G7	up	1.530232886	1.622678202
IRAK3	up	2.202326679	1.422521109
AIF1	up	1.795410607	1.543142202

RGS1	up	1.338061994	1.922301755
P2RY13	up	1.81062925	1.682165881
IFI30	up	1.160135961	1.506883061
BCL2A1	up	1.839632663	1.755854445
CLEC7A	up	1.730021778	1.161184807
PMAIP1	up	1.249651825	1.249651825
FGL2	up	1.567077011	1.655018345
C15orf48	up	1.929688047	1.272815491
OGN	up	2.137645005	1.345305027
PLA1A	up	1.022353398	1.178920078
LILRB2	up	1.56287516	1.482681701
MNDA	up	1.505300369	1.417373452
S100A8	up	1.925739845	1.417006456
ANKRD22	up	1.516134532	1.485198263
IGSF6	up	1.410497179	1.248622072
IL18	up	1.243459543	1.356593173
NLRC4	up	1.073539929	1.497579305
S100P	up	2.182351971	1.257035563
TMEM176A	up	1.203242225	1.078869797
IGFBP7	up	1.758303488	1.367246886
FBN2	up	1.429499919	1.1728721

NCF2	up	1.622328815	1.058987131
MS4A6A	up	2.076485285	1.350919143
GPR84	up	1.25185155	1.443732283
AOAH	up	1.349929302	1.054392035
NME8	up	1.69269993	1.444965312
IGFBP3	up	1.252110439	1.088326748
HP	up	1.073617855	1.058809549
TFEC	up	1.847355693	1.392168641
EPPK1	down	-1.046348602	-1.320141598
BCL9	down	-1.108816601	-1.23530129
SPON1	down	-1.06601844	-1.051945782

Table S2 KEGG enrichment analysis of common DEGs

Category	Term	Count	Padj	FDR
KEGG_PATHWAY	Leukocyte transendothelial migration	5	0.000036	0.015058451
KEGG_PATHWAY	Lipid and atherosclerosis	6	0.00007	0.015058451
KEGG_PATHWAY	Neutrophil extracellular trap formation	5	0.0004	0.061200362
KEGG_PATHWAY	Leishmaniasis	3	0.0022	0.199290381
KEGG_PATHWAY	Fluid shear stress and atherosclerosis	4	0.0012	0.157117222

Table S3 GO enrichment analysis of common DEGs

Category	Term	Count	Padj	FDR
GOTERM_BP_DIRECT	GO:0050729~positive regulation of inflammatory response	6	0.0000013	0.008406887
GOTERM_BP_DIRECT	GO:0032731~positive regulation of interleukin-1 beta production	5	0.0000019	0.008406887
GOTERM_BP_DIRECT	GO:0006954~inflammatory response	9	0.0000055	0.008406887
GOTERM_BP_DIRECT	GO:0016046~detection of fungus	2	0.0000098	0.186655078
GOTERM_BP_DIRECT	GO:0051092~positive regulation of NF-kappaB transcription factor activity	6	0.000012	0.018181308
GOTERM_BP_DIRECT	GO:0032755~positive regulation of interleukin-6 production	5	0.000019	0.031306025
GOTERM_BP_DIRECT	GO:0002526~acute inflammatory response	3	0.000033	0.08080426
GOTERM_BP_DIRECT	GO:0042102~positive regulation of T cell proliferation	4	0.000053	0.05542875
GOTERM_BP_DIRECT	GO:0002755~MyD88-dependent toll-like receptor signaling pathway	3	0.000044	0.082796066
GOTERM_BP_DIRECT	GO:0043065~positive regulation of apoptotic process	7	0.000081	0.041144867
GOTERM_CC_DIRECT	GO:0005576~extracellular region	22	7.80E-08	3.63E-05
GOTERM_CC_DIRECT	GO:0005615~extracellular space	19	0.0000018	0.000402
GOTERM_MF_DIRECT	GO:0005201~extracellular matrix structural constituent	5	0.000076	0.096005502

TableS4 Top 20 hub genes in six algorithms

EPC	Degree	Stress	MCC	MNC	Closeness

TLR8	TLR4	MMP9	TLR8	TLR4	TLR4
TLR4	TLR8	TLR4	MS4A7	TLR8	TLR8
CYBB	LILRB2	CYBB	LILRB2	LILRB2	CYBB
LILRB2	CYBB	TLR8	TLR4	CYBB	LILRB2
CD163	CD163	CD163	IGSF6	CD163	CD163
MNDA	MMP9	LILRB2	CYBB	MNDA	MMP9
IGSF6	MNDA	NCF2	CD163	MMP9	MNDA
MMP9	IGSF6	MS4A6A	MNDA	IGSF6	IGSF6
FGL2	IL18	IL18	FGL2	FGL2	IL18
IL18	FGL2	BCL2A1	MS4A6A	IL18	NCF2
AIF1	NCF2	IGSF6	IL18	NCF2	VCAM1
NCF2	MS4A6A	MNDA	CLEC7A	VCAM1	FGL2
VCAM1	VCAM1	POSTN	MMP9	MS4A6A	AIF1
CLEC7A	AIF1	VCAM1	AIF1	CLEC7A	S100A8
MS4A6A	CLEC7A	S100A8	AOAH	AIF1	MS4A6A
S100A8	S100A8	F13A1	P2RY13	PECAM1	PECAM1
CD1D	PECAM1	AIF1	NCF2	GZMB	CLEC7A
MPEG1	GZMB	MS4A7	CD1D	CD1D	MPEG1
GZMB	CD1D	PECAM1	MPEG1	S100A8	GZMB
PECAM1	MPEG1	FGL2	S100A8	MPEG1	CD1D

TableS5 Top 20 hub genes in six algorithms

Gene	Description
TLR8	toll like receptor 8
LILRB2	leukocyte immunoglobulin like receptor B2
TLR4	toll like receptor 4
IGSF6	immunoglobulin superfamily member 6
CYBB	cytochrome b-245 beta chain
CD163	CD163 molecule
MNDA	myeloid cell nuclear differentiation antigen
FGL2	fibrinogen like 2
MS4A6A	membrane spanning 4-domains A4A
IL18	interleukin 18
MMP9	matrix metallopeptidase 9
AIF1	allograft inflammatory factor 1
NCF2	neutrophil cytosolic factor 2
S100A8	S100 calcium binding protein A8

TableS6 Candidate drugs (top ten) identified from gene−drug interaction enrichment analysis

Name	Ajusted P-value
9,12-Octadecadienoic acid CTD 00007296	4.50E-05
dexamethasone CTD 00005779	8.97E-05
Phorbol 12-myristate 13-acetate CTD 00006852	1.35E-04
acetoacetic acid BOSS	1.77E-04

NADP BOSS	1.81E-04
Chromium(VI) CTD 00002830	4.14E-04
ACROLEIN CTD 00005313	4.73E-04
benzo[a]pyrene CTD 00005488	5.29E-04
sulfaguanidine PC3 UP	6.20E-04
arachidonic acid CTD 00007139	6.90E-04

TableS7 The binding sites and energies for key drug targets were evaluated through AutoDock calculations

Drug targets	Binding energy
**IGSF6**	
Octadecadienoic_acid	-3.4
acetoacetic_acid	-3.3
Acrolein	-2.3
arachidonic_acid	-3.4
Benzo	-6.5
dexamethasone	-5.8
NADP	-6.6
Phorbol_12_myristate_13_acetate	-4.9
Sulfaguanidine	-5.1
**MMP9**	
Octadecadienoic_acid	-4.3

acetoacetic_acid	-3.7
Acrolein	-2.4
arachidonic_acid	-4.1
Benzo	-6.7
dexamethasone	-6.6
NADP	-7.4
Phorbol_12_myristate_13_acetate	-6
Sulfaguanidine	-5.9
**MNDA**	
Octadecadienoic_acid	-3
acetoacetic_acid	-3.2
Acrolein	-2.1
arachidonic_acid	-3.2
Benzo	-5.5
dexamethasone	-5.5
NADP	-5.6
Phorbol_12_myristate_13_acetate	-4.7
Sulfaguanidine	-4.4
**NCF2**	
Octadecadienoic_acid	-3.6
acetoacetic_acid	-3.1

Acrolein	-2.3
arachidonic_acid	-4.3
Benzo	-6.1
dexamethasone	-5.5
NADP	-5.7
Phorbol_12_myristate_13_acetate	-4.6
Sulfaguanidine	-4.5
**S100A8**	
Octadecadienoic_acid	-4.2
acetoacetic_acid	-3.1
Acrolein	-2.4
arachidonic_acid	-3.9
Benzo_a_pyrene	-7.7
dexamethasone	-6
NADP	-7
Phorbol_12_myristate_13_acetate	-5.2
Sulfaguanidine	-5

TableS8 Basic information for GSE datasets

ID	GSEnumber	Platform	Samples	Disease
1	GSE66360 discover cohort	GPL570	21patients and 22 controls	AMI
2	GSE40611	GPL570	17patients and 18 controls	pSS
3	GSE66360 validation cohort	GPL570	28patients and 28controls	AMI
4	GSE7451	GPL570	10patients and 10controls	pSS
